# The aetiology and burden of myeloproliferative neoplasms in the United Kingdom: the MyelOproliferative neoplasmS: an In-depth case-control (MOSAICC) study protocol

**DOI:** 10.1186/s12885-023-11483-0

**Published:** 2023-12-07

**Authors:** Nouf Abutheraa, Emma-Louise Tarburn, Charlene M. McShane, Andrew Duncombe, Mary Frances McMullin, Lesley Ann Anderson

**Affiliations:** 1 Aberdeen Centre for Health Data Science, Institute of Applied Health Science, School of Medicine, Medical Science and Nutrition, University of Aberdeen, Aberdeen, UK; 2 School of Medicine, Dentistry and Biomedical Sciences, Centre for Public Health, Queen’s University Belfast, Belfast, Northern Ireland UK; 3 University Hospitals Southampton NHS Trust and Hon., University of Southampton Medical School, Southampton, UK; 4 Centre for Medical Education School of Medicine, Dentistry and Biomedical Sciences, Queen’s University Belfast, Belfast, Northern Ireland UK

**Keywords:** Myeloproliferative neoplasms, Genetic mutation, Haematological malignancies, Environmental exposure, Occupational exposure, Aetiology, *JAK2* mutation

## Abstract

**Background:**

Myeloproliferative neoplasms (MPNs) are a group of haematological malignancies that affect approximately 8 people in every 100,000 individuals in the UK. Little is known about the aetiology of MPNs, as previous studies have been hampered by small sample sizes, thus it is important to understand the cause of MPNs in a larger study to identify prevention strategies and improve treatment strategies. This study aims to determine environmental, lifestyle, genetic and medical causes of MPNs and to assess the relevance of occupational carcinogen exposures and quality of life impacts.

**Methods:**

A UK-wide case-control study of 610 recently diagnosed MPN patients (within 24 months) receiving clinical care at 21 NHS study sites in Scotland, England, Wales and Northern Ireland and 610 non-blood relative/friend controls is underway. Data on occupational and residential history, medical and environmental factors, and quality of life are being collected from the participants via a structured interview and self-complete questionnaires. Clinical data is being provided by the clinical team. Blood, saliva and toenail samples are also being collected for genetic and elemental analysis. Adjusted odds ratios (ORs) and 95% confidence intervals (95%CI) will be calculated using a *p* < 0.05 to investigate potential risk factors for the MPN clinical and genetic subtypes, and further analyses will be conducted based on the type of data and outcome of interest at a later stage.

**Discussion:**

The study design is most effective for investigating the aetiology of rare diseases. The study will enable identification of potential causes of MPNs through in-depth assessment of potential risk factors with potential for longer follow-up of a number of outcomes.

**Supplementary Information:**

The online version contains supplementary material available at 10.1186/s12885-023-11483-0.

## Background

Myeloproliferative neoplasms (MPNs), previously termed myeloproliferative disorders (MPDs), are a group of haematological malignancies that affect the myeloid cells in the bone marrow [1]. The classic MPNs include polycythaemia vera (PV), essential thrombocythaemia (ET) and primary myelofibrosis (PMF) [2]. Collectively the conditions affect approximately 7.9 per 100,000 individuals in the UK [3], with PV and ET more common than PMF [1, 4, 5].

MPNs are generally indolent disorders; treatment decisions are based on risk stratification with the primary treatment objective of preventing complications [6]. While 5-year relative survival has been reported to be high (89.9–76%) [7, 8], quality of life (QoL) can be significantly impaired. A USA study reported 81% of MPN patients experienced fatigue, 35% needed assistance with their daily living and 11% reported a medical disability as a result of their MPN [9].

MPNs are associated with somatic mutations including *Janus kinase 2* (*JAK2)*_*v617F*_ [10–12] present in almost all PV and approximately half of ET and PMF patients [13], and *Calreticulin* (*CALR*) mutation, present in 20–35% of ET and PMF patients [14]. *MPL* mutations are also present in 4% of ET and 5–9% in myelofibrosis patients [15]. Emerging data suggests that these acquired mutations have distinct clinical phenotypes with prognostic significance [16]. A recent study suggested that *JAK2*_*v617F*_ mutations may occur early in life *or utero* but that environmental factors may influence progression to cancer [17].

Cancer incidence is predicted to double by 2070 primarily due to an ageing population [18]. It is critical to identify ways to prevent MPNs and to improve diagnosis and treatment in order to reduce the burden of disease. A systematic review by our team in 2012 identified a higher mortality rate for people with MPNs with certain occupations such as poultry workers (n = 2 myelofibrosis patients), and petroleum refinery workers (n = 2/4 myelofibrosis and PV patients respectively) [19]. Benzene exposure was also associated with a 50-fold increase risk of developing an MPNs (n = 9) based on 1 study [19]. A pilot study (106 MPN; 120 controls) conducted during 2013 to 2014 by our team found associations between MPNs and smoking, obesity and CT scans [20]. To date, prior studies have been hampered by small sample sizes making it difficult to draw any conclusion or association. In general, there is also the challenge of using self-reported occupational tasks and exposure history which has a low agreement (50.6–67%) when validating the self-report with the company/employer data [21]. This led to the development of Job-exposure matrices (JEMs) in an effort to provide a more accurate recall of occupational carcinogen exposure using expert assessment [21]. Taking into consideration the above limitations, a larger robust case-control study is needed to understand these factors and their impact.

The MOSAICC study (MyelOproliferative neoplasmS: An In-depth Case-Control) is an ongoing UK-wide case-control study which aims to improve understanding of the aetiology of MPNs, prevention and mechanisms of disease and impact of MPNs on quality of life.

### Aims

A UK wide case-control study is being undertaken to: (1) determine environmental, lifestyle and medical causes of MPNs, (2) assess the relevance of occupational carcinogen exposures, (3) identify epidemiological signatures associated with MPN mutations, (4) develop a comprehensive understanding of the symptomatology and psychosocial impact of MPNs, (5) explore how potential risk factors may influence patient prognosis, (6) assess exposure to trace elements in toenail samples and assess further exposure via occupational assessment and geospatial analysis of occupational and residence history (7) undertake genetic assessment of MPNs patients and controls using blood and saliva samples.

## Methods

### Study design & setting

MPN patients receiving care at one of the 21 participating NHS study sites across Scotland, England, Wales and Northern Ireland are being recruited to participate in a case-control study. A substudy on the role of vitamin D levels in MPN patients is also being undertaken in 8 sites.

### Recruitment timeframe

The MOSAICC study opened its first site in August 2020. Recruitment was then halted during the early stages of the COVID-19 pandemic. Recruitment restarted in October 2021 with an initial recruitment duration of 26 months. The recruitment timeframe has been subsequently extended for 12 months until December 2024 due to ongoing recruitment challenges relating to ongoing COVID-19 impacts on the health service (affecting workforce capacity) and lower than anticipated numbers, and this is subject to change following the required ethical amendment if needed.

### Characteristics of participants

The study aims to recruit approximately 610 MPN patients (cases) and up to 610 non-blood relatives or friends (NBR/F) i.e., controls.

**Case inclusion criteria**:

1. Have a clinically confirmed MPN diagnosis (PV, ET or PMF); based on the WHO and the British Society for Haematology definition [22–25] - [see Additional file 1].
2. Have been informed that they have an MPN by their clinical team.
3. Diagnosed within the previous 24 months. To capture their current lifestyle before any changes occur as a result of their diagnosis which could make it difficult to identify the temporality of the association between the risk factors under investigation and the conditions being investigated.
4. Aged 18 years or over.
5. Capable of giving informed consent as determined by the treating clinician.
6. Physically and cognitively capable of completing questionnaires as determined by the treating clinician.
7. Capable of completing the telephone interview in English language as determined by the treating clinician.

Patients will be excluded if they are under 18 years old, incapable of giving informed consent, physically or cognitively incapable of completing the questionnaires and/or telephone interviews, too ill to participate or not expected to live more than 2 months as determined by the treating clinician. Patients who cannot complete the study requirements in English language are not eligible to participate.

**Control**: NBR/F’s with no known diagnosis of MPNs will be recruited by participating MPN cases with a 1:1 ratio. Controls will need to be of the same sex and aged no more than 10 years younger (must be aged 18 years or older) or 10 years older than the case.

Using NBR/F’s as controls were chosen as NBR/F controls are known to be more convenient and less expensive to recruit and useful for rare diseases where patients are referred to specialist centres [26]. Our MOSAICC pilot study found that NBR/F controls were more likely to participate than GP controls (74% vs. 17% participated in the pilot study, respectively) [27]. While it is acknowledged that there are limitations with this method, such as leading to the closer matching of the case and control populations, similar characteristics would attenuate observed associations rather than create erroneous associations [28].

### Study pathway

A standard practice is followed, and further details are available about the study pathway [see Additional file 2].

### Data collection tools

#### 1. Initial patient Assessment Form (cases only)

The clinical team at each recruitment site assess patient eligibility to participate in the study. This includes completing the study inclusion criteria checklist, recording of patient’s sex, age, type of MPN and time since diagnosis. If a patient does not meet the study criteria, the reason for exclusion is listed. In addition, information about the discussion around the study and reminder calls will be collected. Finally, if the patient was given a study package the invitation ID will be documented.

#### 2. Consent form

Both cases and controls are required to complete a consent form and return to the MOSAICC study team. Once received by the MOSAICC study team, the consent form is checked and only co-signed once a study team member has made contact with the participant and reconfirmed consent verbally. A password-protected scanned copy of the patient consent form is sent to the clinical site for inclusion in their clinical records. All participants receive a copy of their consent form to retain via post.

#### 3. Contact information sheet

Data will be collected on the participant’s name, address, date of birth, contact number and email. This sheet will also have information on the most suitable time for the participant to receive a telephone call from the research team.

#### 4. Occupation/residence calendar

An occupation and residence calendar will be used to gather information about participants’ work and residential history. The occupation calendar includes the job title, years started, the year finished, the number of hours per week, the number of weeks per year and the city or town for each job. The residence calendar includes the postcode or name of the street and city or town of residence. Participants will be asked to report only occupations and residences of a minimum of 6 months duration.

#### 5. Telephone interview

The telephone interview questions were co-designed with patients, clinicians, and researchers and informed by a pilot study [19, 29] and MPN Voice, one of the charitable funders, asking patients what they thought should be included in the questionnaire. The questionnaire includes a collection of information on demographic data, medical history, occupational assessment, and lifestyle.

The interview will be conducted remotely using Microsoft Teams Telephony with data entered directly into Qualtrics^XM^, an online questionnaire platform, by the interviewer. Participants will be asked for additional verbal consent at the time of the telephone interview to have their interview recorded for quality control purposes both before and after (if agreeable) the recording is activated. The voice recordings will be held for the duration of the study to enable a random 5% check for completion and quality by the research team then they will be deleted. Any individuals who are hard of hearing will be asked to have someone with them who will be able to assist in interviewing if needed. For those who are deaf and can answer using a text phone a text-reply system will be utilised.

The occupational assessment will be done using the Occupational Integrated Database Exposure Assessment System (OccIDEAS) – a web-based software that was developed by an expert group of occupational epidemiologists and a panel of occupational hygienists that use Job-Specific Modules (JSMs) to input data on exposure [30]. It will help map each job to the appropriate module within the guided questionnaire to obtain more detailed information on potential exposures. Participants will be allocated specific questions relevant to each job role, for example, there is a JSM for carpenter and another JSM for health professional which could improve the reporting of occupational exposures [30]. A complete description of OccIDEAS is available elsewhere [30].

### Reminder

A reminder text message/e-mail will be sent (if requested) to participants to remind them about the interview call.

#### 6. Quality of life questionnaires

To assess symptom burden and quality of life the 17-item MPN-symptom assessment form (SAF) questionnaire, Short-Form 12v2® and EQ-5D-5L is used. A bespoke COVID-19 questionnaire has been included to evaluate the recent impact of the pandemic on individual QoL. In line with feedback from patients we have also included an open-ended question about traumatic experiences and their impact on the participant’s life.

The MPN-SAF was first developed in 2009 as the first patient-reported outcome measure (PROM) tool for myelofibrosis symptoms [31]. It was redeveloped in 2011 to include PV and ET symptoms [32]. In 2012 a shorter version of the questionnaire was developed, the MPN-SAF total symptom score (TSS), covering the 9 most common symptoms [33], with more recent updates featuring 7 symptoms [9].

The Short-Form 12v2® is the most used questionnaire to assess health-related QoL [34]. It was originally designed by the Medical Outcomes Study (MOS) 36-item Short-Form Health Survey SF-36 which was shortened to make it more practical. The Short-Form 12V2® covers eight health domains (the same ones as the SF-36) using just 12 questions and has been shown to have good reliability [35]. The EQ-5D-5 L is a standardised questionnaire developed by the EuroQol group to measure QoL. It provides a good overall measure of self-reported QoL across the following domains: self-care, usual activities, pain/discomfort and anxiety/depression [36]. Therefore, we will be using several questionnaires to capture a variety of data and allow comparison with control QoL.

If a participant (control or patient) indicates something of serious concern such as harm to themselves or others in their response to the QoL questionnaires, as a duty of care, the study chief investigator at the University of Aberdeen will contact them to provide information on support services. For blind participants, the QoL questionnaires and any other written form can be completed by phone with a member of the research team.

#### 7. Biological samples

The following samples will be collected from all participants who consented to provide the named sample below on their consent form.

### Blood samples

A total of 7.5ml of blood samples using **2**-DNA PAXgene blood tubes and **1**-RNA PAXgene blood tube per participant are to be withdrawn by the clinical team.

The tubes will be placed in a Royal Mail Safebox™ with absorbent paper to be sent to the University of Aberdeen where the sample will be stored at a -20 °C freezer for a minimum of 48 h before being transferred to a -80 °C freezer for long term storage.

- **A phlebotomy record sheet** will be completed by the person drawing the blood to record the time and date the sample was taken. This sheet will include space for the MOSAICC study team to record the date and time that the sample was received, transferred to the − 20^o^C and − 80^o^C freezer and any details regarding the sample conditions upon receipt from the hospital.

Sites with the Vitamin D sub-study will process an additional Vitamin D3 sample for analysis for patients only using their standard blood tube (6ml) that will be sent to their on-site laboratory for analysis. The result will be recorded on the patients’ Clinical Report Form (CRF).

### **Whatman® FTA card**

Only if a participant cannot attend a phlebotomy appointment, an alternative dry blood spot collection will be offered.

### Saliva samples

Participants will be sent the saliva collection kit along with an instruction leaflet and a saliva pressure-tested transport pack that contains an absorbent pad with a secure sealing strip. The pressure-tested transport pack will be placed in a padded envelope and placed in the freepost envelope with the other documents for safely shipping the saliva sample back to the research team.

Saliva samples (2mL) using the GeneFiX™DNA collection tube remain stable for several years [37], and therefore will be stored at room temperature before batch transfer to a -80 °C freezer.

### Toe-nail specimens

Participants will be asked to wash their feet and remove any nail varnish before cutting their toenails from each great toe. Samples will be placed in a sample collection bag and posted back to the study team. The samples will be stored in a filing cabinet until they are processed to investigate elemental analysis.

#### 8. Clinical notes (cases only)

Permission to access hospital notes for patients (cases) only is requested on the consent form. An electronic and/or paper-based proforma for the CRF will be completed by the clinical team.

#### 9. Informing GPs of inclusion in the study (cases only)

GPs will be informed of the inclusion of their patients in the study (letter from the clinical team) following the receipt of the patient’s informed consent.

### Decline to participate/withdraw

The number of subjects who decline to participate will be documented. Subjects will be given the opportunity to withdraw from the study at any time and the data/samples destroyed if requested with a record maintained of whether this person was a case/control, their age, sex and reason for withdrawing from the study (if given).

### Sample size

In the EpiLymph study, which investigated occupational exposures and the risk of lymphoma, the proportion of controls exposed to any solvent was 29.6% [38]. Based on 610 cases and controls there will be more than 97% power to detect an OR of 1.6 between MPN cases and controls based on a 29.6% prevalence of solvent exposure in the controls. In the same study, benzene exposure was reported in 5.7% of controls [39]. Given this prevalence, the study will have more than 96% power to detect an OR of 2.2 between cases and controls.

### Data management

REDCap will be used for data entry. REDCap is a browser-based, metadata-driven software for designing research databases by providing collaborative access to data via user authentication and role-based security [40]. It allows the clinical team to have access to their data and limits them from having access to the whole cohort of participants. REDCap also facilitates data validation and completion which is used within the study team between and within universities [40].

The core research team [subject to regulatory approvals] have access to enter data. External researchers and students will only be provided with pseudo-anonymised data for analysis purposes.

Biological samples: If the participant supplies saliva, blood and/or toenail samples for the study, they will be stored in compliance with the Human Tissue Act (Northern Ireland) and the Grampian Biorepository (NHS Grampian).

Data collected from participants will be retained for the period of the study and kept in the University of Aberdeen server/premises for 10 years as per university policy. The dataset will be saved on the University of Aberdeen Shared Drive, and the owner of the dataset is the study CI (LAA).

### Data analysis

Access to the identifiable data will be managed by authors LA & CMcS and access to anonymous data during the analysis process using *R* Programming language or Stata will be handled by the study team and other staff or students if required. Controls will not be individually matched to cases (frequency matching will be used). The analysis will compare cases and controls in all aspects of the data collected, for example, to assess the level of carcinogenic exposure from some occupations and other potential risk factors we will compare between controls and all cases combined, PV, ET and PMF separately, *JAK2* positive / *JAK2* negative, and *CALR* positive / *CALR* negative using multivariate logistic regression/ regression analyses as appropriate with adjusting for potential confounders. Adjusted odds ratios (ORs) and 95% confidence intervals (95%CI) will be calculated using a *p* < 0.05 to be considered as a statistically significant value. Further analysis for questionnaires will be considered as per published methodologies, [41, 42], and occupational exposures will be analysed using the OccIDEAS platform to assess carcinogenic exposure which will be classified as exposed and not exposed, these categories will be compared using Chi-Square (X^2^) test between patients and controls. The toenail sample will be digested (microwave digestion in duplicate) and then analysed using mass spectrometry, for both blood and saliva samples gene-environment interactions will be investigated in a genome-wide association study (GWAS), and a subset (n = 50) will be analysed using Long Read-Sequencing to read through complex regions and thus enhance the detection of variants identified using GWAS.

## Discussion

Whilst genetic factors predispose the development of MPNs, evidence suggests it is likely that environmental and/or lifestyle factors have an impact on the development of clinical disease. Previous epidemiological studies investigating the aetiology of the MPNs have been limited by small sample size, heterogeneity in study design, and the inability to investigate sub-type or mutational specific risk [19]. An initial exploratory case-control study was conducted to assess the feasibility of a large, multi-centre epidemiological study of the MPNs, with the results informing the optimal methodological approach for this patient group. The MOSAICC pilot study compared case ascertainment, control recruitment methods, the evaluation of exposure information, symptom assessment tools and the collection, storage and analysis of biological materials, leading to a number of significant publications [4, 19, 20, 43–46].

A 2010 systematic literature search identified a number of risk factors for MPN development and informed the data collected in the pilot [19]. Invitation to participate in the MOSAICC pilot study was sent to 538 individuals, with the aim to recruit 300 participants (100 cases and 200 controls). The study recruited 233 of the 538 invited (43%), and 78% of the intended target. Response rates were higher in cases (67%) than controls (34%) [20]. MPN patients and controls provided information that included recruiting site (Belfast City Hospital, Belfast or University Hospital Southampton NHS Foundation Trust, Southampton) sex, age, Jewish ancestry, childhood socioeconomic position, pack-years smoking, birth order and number of siblings, alcohol consumption, pre-existing medical conditions, number of dental amalgam fillings and hair dye use. An occupational carcinogen exposure risk assessment was conducted using the OccIDEAS platform, and QoL and symptom burden was assessed using validated questionnaires.

Despite being a pilot, the study recruited more cases and controls than any other case-control study of the MPNs to date providing sufficient data to assess environmental, medical and lifestyle factors associated with MPN. MPN patients were more likely than controls to have been raised in a household where the main occupation of the head of the household reflected a lower socioeconomic position (OR 2.30, 95%CI 1.02–5.18) [20]. Current cigarette smoking was more common in MPN cases than controls, with a statistically significant elevation observed in PV cases (OR 3.73, 95% CI 1.06–13.15). ET cases were more likely than controls to be obese (OR 2.59, 95% CI 1.02–6.58) [20], however this association was not observed for other MPN subtypes. No significant associations were observed for hair dying, metal or synthetic implants, piercings, or tattoos previously identified in other case-control studies of MPN [20].

MPN-SAF, a reliable and validated tool for the assessment of MPN symptoms [47] was provided to cases and controls. MPN cases had a significantly higher symptom score than controls in 25 out of 26 parameters measured, with fatigue the most common symptom reported. This was the first time that MPN-SAF scores were assessed in a control group, and strengthens the understanding of the symptom burden experienced by MPN patients [44].

In assessing the feasibility for collection and storage of biological samples in epidemiological studies, cases and controls were asked to provide two biological samples: a blood sample and a self-collected saliva sample. The mean DNA yield was sufficient for genetic analyses for both specimen types, however significantly higher for blood (659.18 ng/µL) than saliva (275.79 ng/µL) [43]. Of the participants, 89% provided a blood sample and 93% provided a saliva sample demonstrating that it was achievable to obtain biological samples from the majority of participants in case-control studies [43], and thereby build a biobank for future molecular epidemiological studies with controls.

However, whilst the study identified a number of significant associations between explored risk factors and MPN development, the small sample size (106 MPN; 120 controls) limited the ability to assess sub-type specific risk indicating that a systematic, multicentre study using the pilot-defined methodologies was still necessary to contribute to the body of evidence in MPN epidemiology [48].

The MOSAICC study will be significantly larger than any other case-control study of the MPNs to date and will assess sub-type and/or mutational specific risk factors for MPN development, in addition to symptom severity and quality of life across subtype/mutational groups. Although case-control studies are the most effective methodology for investigating risk factors for rare cancers, they are often limited by recall bias. Strategies to minimise for this have been implemented into the study protocol and whilst these strategies can help reduce the effects of this bias, the authors acknowledge that differential recall bias may still occur.

The pilot study investigated control recruitment via two methods; recruitment from GP surgeries and NBR/F invited directly by case participants, finding little variation between control groups in the majority of assessed risk factors [20]. It was established the NBR/F controls were less costly and more convenient to recruit, and whilst it is acknowledged that this method may lead to closer matching of cases and controls (i.e., cases recruiting co-workers, for example), age and sex matching has the advantage of attenuating results rather than contributing to erroneous associations. As for the in-depth assessment of job-based exposure, it should still enable the identification of potential exogenous occupational exposures even if friends work within the same company. Even with modest numbers, our pilot study demonstrated significant differences in several parameters under investigation between cases and NBR/F controls despite the likelihood of shared factors [20]. Therefore, strongly associated risk factors are likely to be identified by utilising this approach. Despite this, it has also been considered that the Covid-19 pandemic may have contributed to a reduction in the social circle of many of the case participants, reducing the potential pool of controls available to the case. Control participation rates will be monitored with alternative control recruitment strategies considered. As data analysis will be undertaken on MPN genetic and phenotypic subtypes the same control group will be used enabling a lower control number compared to cases.

Single-cell transcriptomics studies reconstructing the lineage history of the JAK2_*V617F*_ mutation recently identified that it often occurs in a single stem cell decades prior to diagnosis, with mutation acquisition even shown to occur *in utero* [49]. This study provided significant insights into the latency of MPNs but data capture in the MOSAICC study spans the case and control life course enabling identification of important drivers of mutation acquisition.

Questions remain as to what risk factors may drive clinical disease, whether these are modifiable, and what preventative strategies could be put in place for those with a MPN related mutation. The MOSAICC study, which is currently recruiting from 19 sites across the UK, should be larger than any previous work and has the potential to provide significant insights into the epidemiology of MPNs.

### Electronic supplementary material

Below is the link to the electronic supplementary material.

Supplementary Material 1

Supplementary Material 2

## Data Availability

Not applicable.

## Abbreviations

95%CI: 95% Confidence Intervals
BMI: Body mass index
*CALR*: *Calreticulin*
CRF: Clinical Report Form
CRNs: Clinical Research Nurses
ET: Essential Thrombocythaemia
GCP: Good Clinical Practice
GP: General Practitioner
GWAS: genome-wide association study
IPAF: Initial Patient Assessment Form
*JAK2*: *Janus kinase 2*
MPDs: Myeloproliferative Disorders
MPNs: Myeloproliferative Neoplasms
NBR/F: Non-Blood Relative / Friend
OccIDEAS: Occupational Integrated Database Exposure Assessment System
OID: Organisation Information Document
ORs: Odds Ratios
PI: Principal Investigator
PIS: Participant Information Sheet
PMF: Primary Myelofibrosis
PRO: Patient Reported Outcome
PV: Polycythaemia Vera
QoL: Quality-Of-Life
SAF: Symptom Assessment Form
SIV: Site Initiation Visit
