# Supplementary material for: The aetiology and burden of myeloproliferative neoplasms in the United Kingdom: the MyelOproliferative neoplasmS: an In-depth case-control (MOSAICC) study protocol
Source: BMC Cancer. 2023 Dec 7;23:1207. doi: 10.1186/s12885-023-11483-0 (PMC10704614; doi:10.1186/s12885-023-11483-0)
Supplement: Supplementary file 1 — Supplementary Material 1 [file 12885_2023_11483_MOESM1_ESM.docx]

**Additional file 1: Definition of cases**

Patients with PV, ET or PMF will be recruited based on the following diagnostic criteria:

**Polycythaemia Vera**

**Diagnosis requires both criteria to be present**

**A1.** High haematocrit (>0.52 in men, >0.48 in women) OR raised red cell mass (>25% above predicted)

**A2.** Mutation in JAK2

**Essential Thrombocythaemia**

**Diagnosis requires A1-A3 or A1+A3-A5**

**A1.** Sustained platelet count >450 x109/L

**A2.** Presence of an acquired pathogenetic mutation (e.g. in the JAK2 or MPL genes)

**A3.** No other myeloid malignancy, especially PV, PMF, CML or MDS

A4. No reactive cause for thrombocytosis and normal iron stores

**A5.** Bone marrow aspirate and trephine biopsy showing increased megakaryocyte numbers displaying a spectrum of morphology with predominant large megakaryocytes with hyperlobated nuclei and abundant cytoplasm. Reticulin is generally not increased (grades 0-2/4 or grade 0/3)

**Primary Myelofibrosis**

**Diagnostic criteria for primary myelofibrosis: diagnosis requires meeting of 3 major and 2 minor criteria.**

Major Criteria

**A1.** Presence of megakaryocyte proliferation and atypia, usually accompanied by either reticulin and/or collagen fibrosis,

or

in the absence of significant reticulin fibrosis, the megakaryocyte changes must be accompanied by an increased bone marrow cellularity characterized by granulocytic proliferation and often decreased erythropoiesis (ie. prefibrotic cellular-phase disease).

**A2.** Not meeting WHO criteria for polycythemia vera, BCR-ABL1+ chronic myelogenous leukaemia, myelodysplastic syndrome or other myeloid neoplasms.

**A3.** Demonstration of JAK2 V617F or other clonal marker (eg. MPL W515K/L)

or

in the absence of a clonal marker, no evidence that the bone marrow fibrosis or other changes are secondary to infection, autoimmune disorder or other chronic inflammatory condition, hairy cell leukaemia or other lymphoid neoplasm, metastatic malignancy, or toxic (chronic) myelopathies.

Minor Criteria

**B1.** Leukoerythroblastosis

**B2.** Increase in serum lactate dehydrogenase level

**B3.** Anaemia

**B4.** Splenomegaly

**B5.** Leucocytosis
