# Supplementary material for: The aetiology and burden of myeloproliferative neoplasms in the United Kingdom: the MyelOproliferative neoplasmS: an In-depth case-control (MOSAICC) study protocol
Source: BMC Cancer. 2023 Dec 7;23:1207. doi: 10.1186/s12885-023-11483-0 (PMC10704614; doi:10.1186/s12885-023-11483-0)
Supplement: Supplementary file 2 — Supplementary Material 2 [file 12885_2023_11483_MOESM2_ESM.pptx]

## Slide 1
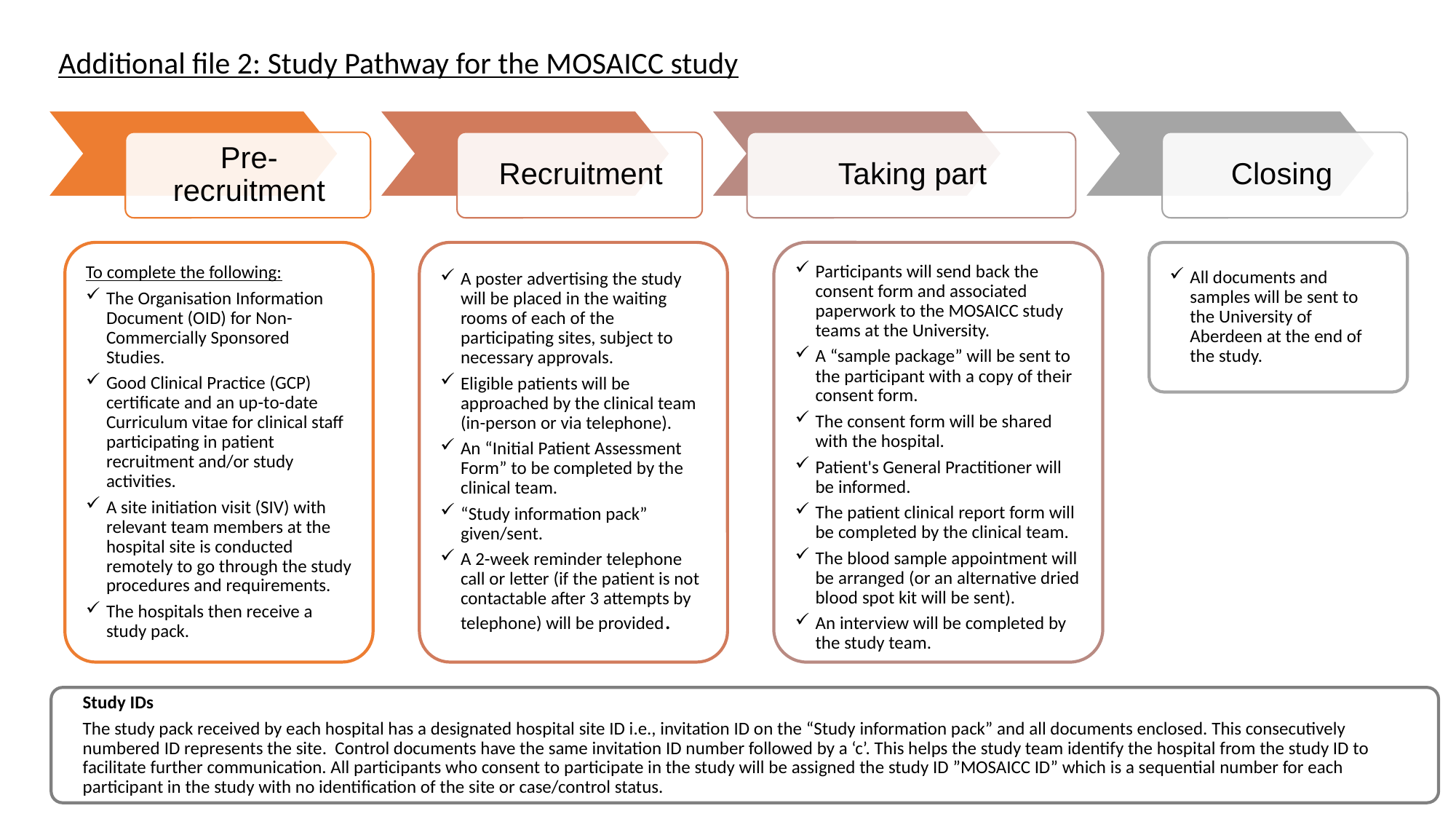

Additional file 2: Study Pathway for the MOSAICC study
To complete the following:
The Organisation Information Document (OID) for Non-Commercially Sponsored Studies.
Good Clinical Practice (GCP) certificate and an up-to-date Curriculum vitae for clinical staff participating in patient recruitment and/or study activities.
A site initiation visit (SIV) with relevant team members at the hospital site is conducted remotely to go through the study procedures and requirements.
The hospitals then receive a study pack.
A poster advertising the study will be placed in the waiting rooms of each of the participating sites, subject to necessary approvals.
Eligible patients will be approached by the clinical team (in-person or via telephone).
An “Initial Patient Assessment Form” to be completed by the clinical team.
“Study information pack” given/sent.
A 2-week reminder telephone call or letter (if the patient is not contactable after 3 attempts by telephone) will be provided.
Participants will send back the consent form and associated paperwork to the MOSAICC study teams at the University.
A “sample package” will be sent to the participant with a copy of their consent form.
The consent form will be shared with the hospital.
Patient's General Practitioner will be informed.
The patient clinical report form will be completed by the clinical team.
The blood sample appointment will be arranged (or an alternative dried blood spot kit will be sent).
An interview will be completed by the study team.
All documents and samples will be sent to the University of Aberdeen at the end of the study.
Study IDs
The study pack received by each hospital has a designated hospital site ID i.e., invitation ID on the “Study information pack” and all documents enclosed. This consecutively numbered ID represents the site. Control documents have the same invitation ID number followed by a ‘c’. This helps the study team identify the hospital from the study ID to facilitate further communication. All participants who consent to participate in the study will be assigned the study ID ”MOSAICC ID” which is a sequential number for each participant in the study with no identification of the site or case/control status.
